# Fast Near‐Infrared Organic Photodetectors with Enhanced Detectivity by Molecular Engineering of Acceptor Materials

**DOI:** 10.1002/advs.202410332

**Published:** 2024-11-27

**Authors:** Wentao Zhong, Xinyuan Wang, Wei Wang, Zhulu Song, Yirong Tang, Bulin Chen, Tingbin Yang, Yongye Liang

**Affiliations:** ^1^ Department of Materials Science and Engineering Southern University of Science and Technology Shenzhen 518055 China; ^2^ Experiment and Practice Innovation Education Center Beijing Normal University Zhuhai 519087 China; ^3^ Core Research Facilities Southern University of Science and Technology Shenzhen 518055 China

**Keywords:** fast response, fused ring electron acceptor, high sensitivity, near‐infrared response, organic photodetectors

## Abstract

Organic photodetectors (OPDs) with a near‐infrared (NIR) response beyond 900 nm are intriguing electronics for various applications. It is challenging to develop NIR OPDs with high sensitivity and fast response. Herein, the acceptor materials of OPDs are tuned to extend detection to ≈1100 nm with improved sensitivity. A new fused ring electron acceptor, ICS (2,2'‐((2Z,2'Z)‐(((4,4‐bis(2‐ethylhexyl)‐4H‐cyclopenta[2,1‐b:3,4‐b']dithiophene‐2,6‐diyl)bis(4‐((2‐ethylhexyl)thio)thiophene‐5,2‐diyl))bis(methaneylylidene))bis(5,6‐difluoro‐3‐oxo‐2,3‐dihydro‐1H‐indene‐2,1‐diylidene))dimalononitrile), is developed with alkylthio thiophene as the bridge, achieving a small bandgap of 1.35 eV while decreasing dark current densities under reverse bias. By further introducing a secondary acceptor of PC_61_ BM, the doping compensation, and unfavored hole injection blocking enable further improvement of detectivity. The PTB7‐Th (Poly[4,8‐bis(5‐(2‐ethylhexyl)thiophen‐2‐yl)benzo[1,2‐b;4,5‐b']dithiophene‐2,6‐diyl‐alt‐(4‐(2‐ethylhexyl)‐3‐fluorothieno[3,4‐b]thiophene‐)‐2‐carboxylate‐2‐6‐diyl]): ICS: PC_61_ BM OPDs deliver a low dark current density of 1.23 × 10^−9^ A cm^−2^, a high peak specific detectivity of 1.09 × 10^13^ Jones at 950 nm under −0.2 V, and a fast response speed with a −3 dB bandwidth of 720 kHz biased under −2 V. The photoplethysmography system with the PTB7‐Th: ICS: PC_61_ BM OPD can reliably monitor heartbeats under 980 nm NIR light. This study promises the development of organic NIR OPDs with high detectivity and fast response by tuning active materials.

## Graphical abstract

A new fused‐ring acceptor, ICS, with alkylthiol thiophene bridges is developed for near‐infrared organic photodetectors, affording expanding spectral response and low dark currents. The incorporation of PC_61_ BM as a secondary acceptor can reduce trap density and serve as a hole barrier, further enhancing detectivity. The PTB7‐Th: ICS: PC_61_ BM devices show not only high detectivity but also fast response.

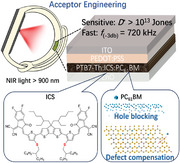

## 1 Introduction

Organic photodetectors (OPDs) utilize cost‐effective and easily scalable manufacturing methods like spin‐coating, printing, and spray deposition, which are applicable to a variety of substrates under mild conditions.^[^ ^1^ ^]^ The mechanical flexibility of OPDs enables them to be lightweight and adaptable to diverse shapes, making them well‐suited for implementation in flexible electronics and wearable technologies.^[^ ^2^, ^3^, ^4^, ^5^ ^]^ In short‐wavelength applications (<700 nm), OPDs have achieved notable advancements by employing donor‐acceptor bulk heterojunction structures derived from organic solar cells (OSCs). These structures optimize light absorption and charge generation, resulting in rapid response times suitable for dynamic imaging and high‐speed data transmission.^[^ ^6^, ^7^, ^8^, ^9^ ^]^ Large‐area, low‐noise flexible OPDs were fabricated, showing high specific detectivity values comparable to silicon photodiodes.^[^ ^10^ ^]^ High cutoff frequencies above 1.5 MHz have also been demonstrated in bulk‐heterojunction systems using a wide bandgap polymer donor and non‐fullerene acceptors.^[^ ^11^ ^]^ Integration with optoelectronic devices such as organic light‐emitting diodes and OSCs offers multifunctional capabilities in compact formats, which are well‐suited for high‐resolution imaging and precise optical communications.^[^ ^4^, ^12^, ^13^, ^14^, ^15^ ^]^

Extending the operational wavelength of OPDs beyond 900 nm has the potential to reduce light scattering and decrease tissue absorption, benefiting applications such as biomedical diagnostics and non‐invasive monitoring.^[^ ^16^ ^]^ However, low bandgap OPDs often face challenges such as high reverse dark currents due to elevated thermionic emission currents, reduced sensitivity owing to inefficient charge separation, and slow response speeds.^[^ ^17^, ^18^, ^19^, ^20^ ^]^ In the early stages of near‐infrared (NIR) OPDs, low bandgap conjugated polymer donors in conjunction with fullerene derivative acceptors (e.g., PC_61_ BM, PC_71_ BM) exhibited low responsivity (<0.2 A W^−1^) partially due to mismatch in energy levels for charge separation. Recent progress has shifted toward employing wide bandgap polymer donors and small bandgap non‐fullerene molecular acceptors with the A‐D‐A structure (e.g., ITIC, IT‐4F, Y6, COi8DFIC), leading to enhanced responsivities (>0.2 A W^−1^) beyond the 900 nm range. Zhu et al. developed PDTIC‐4F and PDTTIC‐4F acceptors, achieving a responsivity of 0.55 A W^−1^ at 900 nm.^[^ ^4^ ^]^ Wang et al. synthesized an acceptor FM2, demonstrating a responsivity of 0.37 A W^−1^ at 880 nm and response up to 1013 nm.^[^ ^21^ ^]^ Despite these advancements, realizing high‐performance NIR OPDs remains challenging, especially at longer wavelength regions, where reduced LUMO‐HOMO offsets hinder charge separation efficiency and responsivity. Response speeds often surpass 100 µs, and dark current densities exceed 10^−5^ A cm^−2^ under reverse bias, underscoring the need for further improvements.^[^ ^22^, ^23^, ^24^ ^]^

Herein, we tune the acceptor materials within the BHJ structure to enhance the performance of OPDs in NIR‐II responsiveness, detectivity, and response. A novel fused ring acceptor, ICS, featuring alkylthio side chains, was designed and synthesized. OPDs based on PTB7‐Th: ICS exhibit lower dark currents than the control with alkoxy side chains. By introducing PC_61_ BM as an auxiliary acceptor into the PTB7‐Th: ICS blend, dark current levels are further reduced without decreasing the corresponding responsivity, thus affording enhanced specific detectivity in the NIR region and an ultrafast response time (<1 µs). Mott–Schottky and X‐ray photoelectron spectroscopy (XPS) depth profiling analyses reveal that incorporating PC_61_ BM not only offers doping compensation but also serves as a hole‐blocking barrier. An advanced Photoplethysmography (PPG) system is developed using the PTB7‐Th: ICS: PC_61_ BM OPD, enabling the real‐time monitoring of heart rate and pulse pressure under weak 980 nm NIR light.

## 2 Results and Discussion

**Figure 1:**
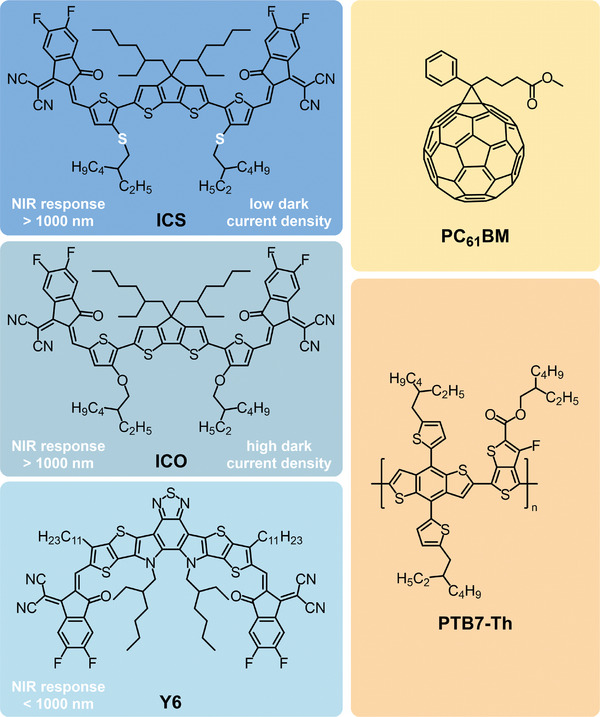
Molecular structures of ICS, ICO, Y6, PC_61_ BM, and PTB7‐Th.

ICO, a low bandgap fused ring acceptor with 3‐((2‐ethylhexyl) oxy)thiophene (3‐EHOT) as bridging units, was reported to show photoresponse up to ≈1100 nm.^[^ ^25^ ^]^ However, its devices generally exhibited relatively high dark currents.^[^ ^14^ ^]^ To overcome this problem, we developed a new electron acceptor called ICS (**Figure** **1**). The key modification in ICS lies in replacing 3‐EHOT in ICO with 3‐((2‐ethylhexyl) thio)thiophene (3‐EHTT), the synthetic routes of ICS depicted in Scheme S1 (Supporting Information). Due to sulfur's lower electronegativity than oxygen, it may enable more effective band gap alignment and improved charge transfer characteristics (Figures S1–S7, Supporting Information).

The molecular geometry was examined using density functional theory (DFT) calculations (Figures S8–S9, Supporting Information).^[^ ^26^ ^]^ ICS possesses a planar molecular backbone structure similar to ICO. The dihedral angle between the thiophene bridge and the donor units is only 3.48 °, slightly larger than that of ICO (0 °). ICS demonstrates a comparable lowest unoccupied molecular orbital (LUMO) energy level to ICO (−3.69 eV for ICS vs −3.64 eV for ICO). However, due to the lower electronegativity of sulfur compared to oxygen, its incorporation into the side chain weakens the electron‐donating ability of the donor unit. Consequently, the highest occupied molecular orbital (HOMO) energy level of ICS drops significantly to −5.57 eV, which is lower than that of ICO (−5.28 eV). The lower HOMO energy level of ICS may enhance the blocking of hole injection from the cathode to the active layer under reverse bias, thus lowering reverse dark currents. Although ICS has a slightly wider bandgap than ICO, the ICS‐based OPDs demonstrate lower reverse dark currents when compared to ICO‐based OPDs (Figure S10, Supporting Information), possibly due to the lower HOMO energy level of ICS for enhanced blocking of hole injection from the cathode to the active layer under reverse bias.

**Figure 2:**
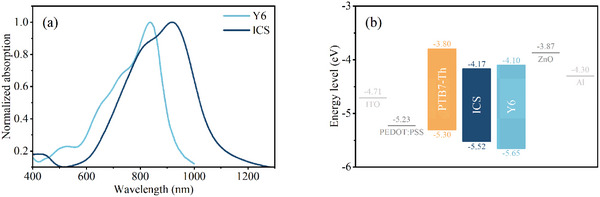
a) The absorption spectra of ICS and Y6 in solid film, and b) the energy levels of the materials.

**Figure** **2**a displays the film absorption spectra of ICS and the typical molecular acceptor, Y6. The ICS film exhibits an absorption peak at 920 nm, which is redshifted by 80 nm compared to Y6, and an absorption onset ≈1100 nm. Cyclic voltammetry (CV) was used to determine the energy levels of HOMO and LUMO for these materials (Figure 2b; Figure S11, Supporting Information). The estimated HOMO/LUMO energy levels of ICS and Y6 are −5.52/−4.17  and −5.65/−4.10 eV, respectively.

**Figure 3:**
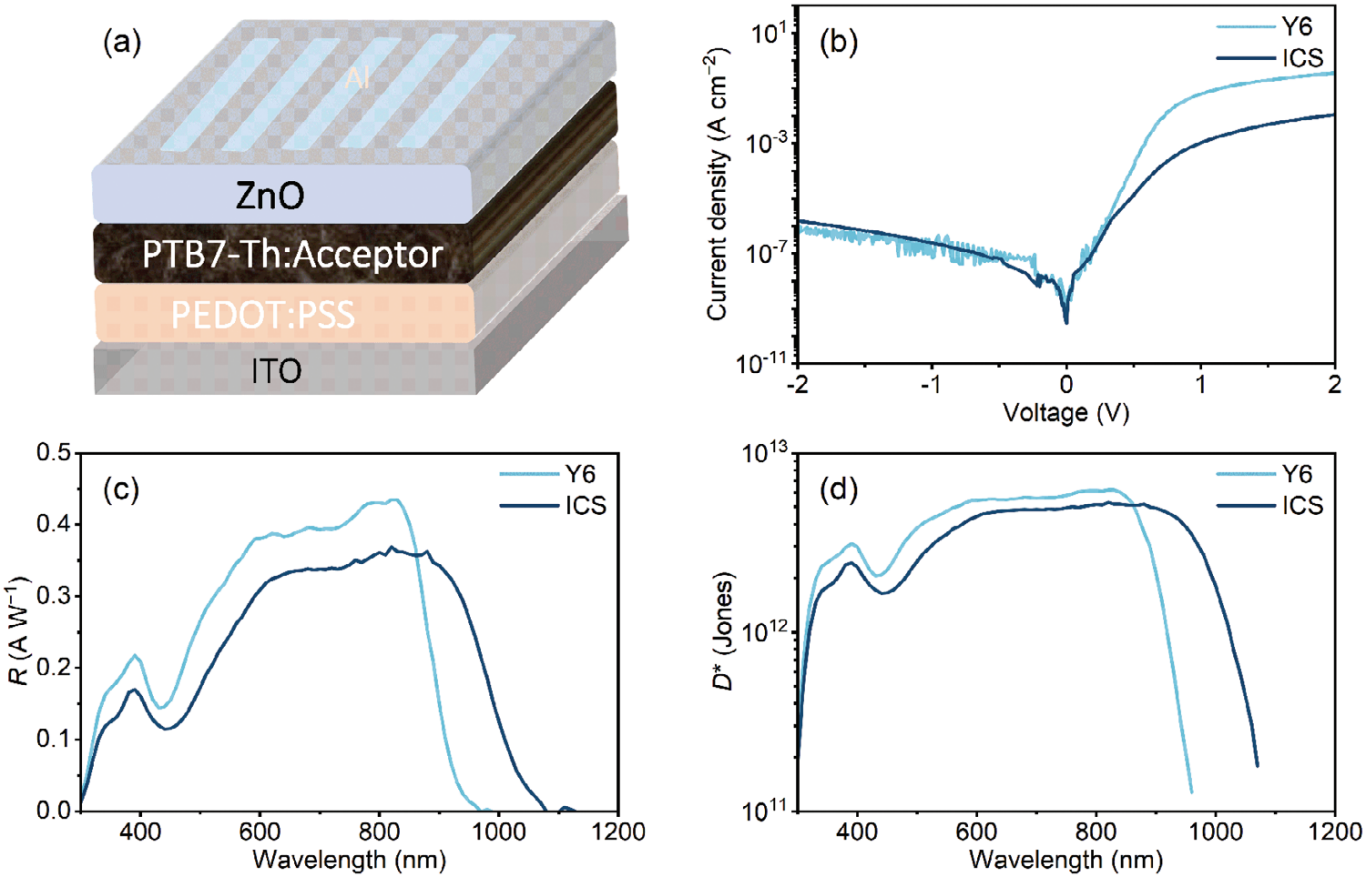
a) The device structure, b) the *J*–*V* curves under dark conditions, c) the responsivity curves, and d) the *D*_sh_^*^ at −0.2 V of the optimized OPD devices utilizing PTB7‐Th: Y6 and PTB7‐Th: ICS.

The ICS film exhibits strong absorption within the near‐infrared spectrum, bearing the potential for the fabrication of NIR OPDs. Considering the energy levels of ICS, a typical electron donor, PTB7‐Th was chosen to be blended with ICS for efficient charge separation. The OPD structure selected is ITO/PEDOT: PSS/PTB7‐Th: ICS/ZnO NCs/Al, as illustrated in **Figure** **3**a. For comparison, OPD devices with Y6 as the acceptor were also constructed as controls.

Figure 3b depicts the semilogarithmic plot of the *J*–*V* curves for OPDs measured under dark conditions. Although ICS has a narrower bandgap than Y6, it still maintains low dark currents. The dark current density of ICS devices reaches 1.0 × 10^−6^ A cm^−2^ at −2 V and further decreases to 2.0 × 10^−8^ A cm^−2^ at −0.2 V, which is comparable to the Y6 OPDs. Responsivity (*R*) was assessed to determine the light detection performance of these devices (Equation S1, Supporting Information). Under a −0.2 V bias, as depicted in Figure 3c, both ICS‐based and Y6‐based devices were evaluated. The ICS device exhibits a photo‐response spanning 300–1100 nm, with *R* values exceeding 0.21 A W^−1^ between 600 and 1000 nm and peaking at 0.38 A W^−1^ at 880 nm. Although ICS devices have slightly lower *R* values in the UV–Vis region than Y6 devices, they demonstrate superior performance beyond 870 nm. ICS achieves a notable *R* value of 0.13 A W^−1^ at 1000 nm. These results indicate that ICS is particularly effective for applications requiring high sensitivity in the deeper NIR range.

To estimate the sensitivity readily, we first calculated the shot noise limited specific detectivity (*D*_sh_ ^*^) of OPDs (Equation S2, Supporting Information), as shown in Figure 3d. The ICS device exhibits a *D*_sh_ ^*^ exceeding 1.0 × 10^12^ Jones from the wavelength of 350 to 980 nm and reaches a maximum *D*_sh_ ^*^ of 1.32 × 10^12^ Jones at 950 nm, as measured at −2 V. The *D*_sh_ ^*^ of ICS devices is comparable to Y6 devices but still suffers from low sensitivity mainly because of the higher reverse dark current densities.

**Figure 4:**
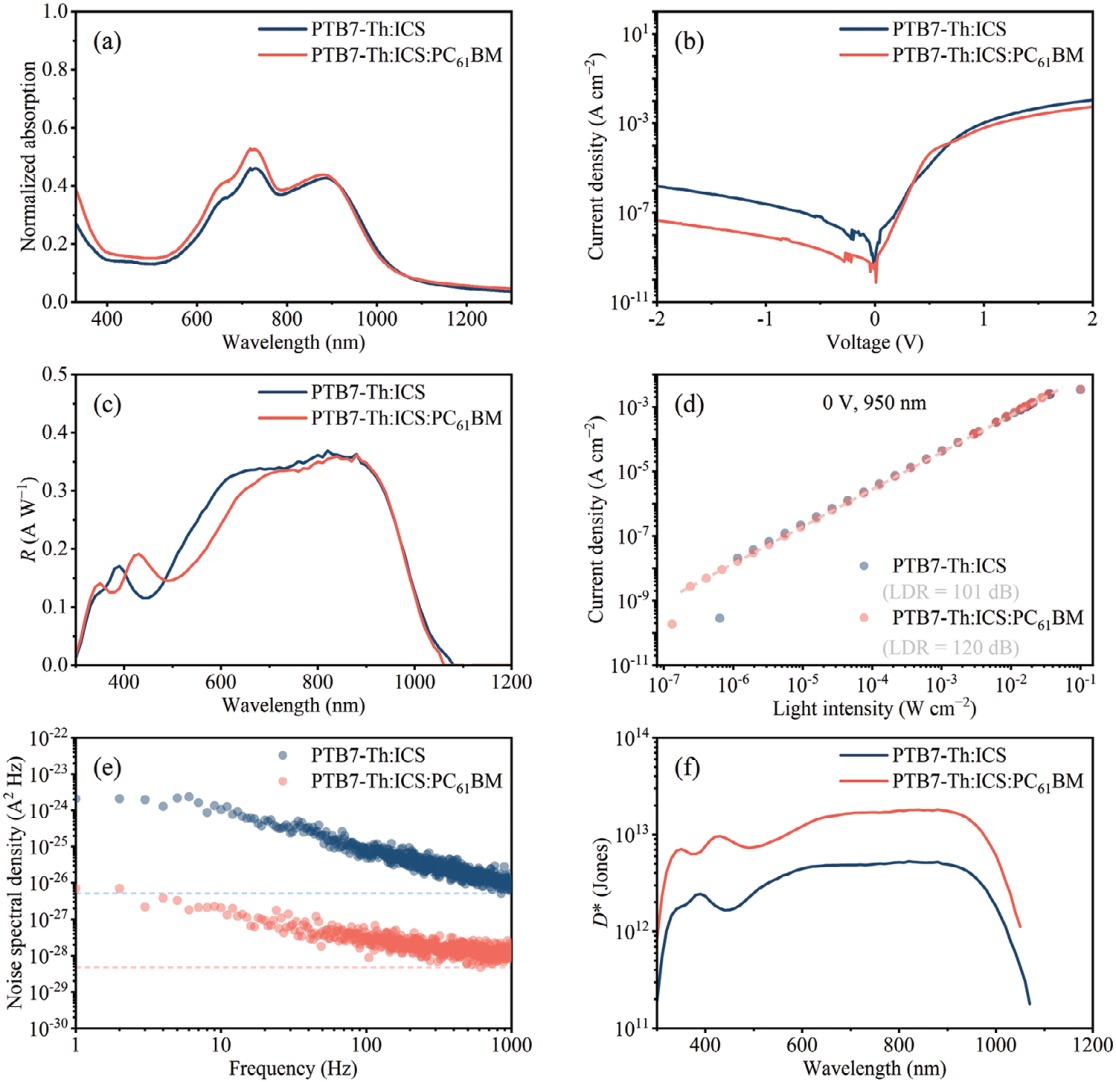
a) Absorption spectra of PTB7‐Th: ICS and PTB7‐Th: ICS: PC_61_ BM in thin films. b) The *J*–*V* curves were measured in darkness. c) *R* curves. d) Linear dynamic range with dashed lines representing linear fitting. e) Noise spectral density at −0.2 V (solid lines show measured noise, and dashed lines indicate calculated white noise). f) the *D* ^*^ at −0.2 V of the optimized OPD devices utilizing PTB7‐Th: ICS and PTB7‐Th: ICS: PC_61_BM.

The low bandgap of ICS could easily result in defect states and traps in the blend, leading to increased device dark current. Integrating the wide bandgap PC_61_ BM as a secondary acceptor could serve as doping compensation for ICS due to their similar LUMO levels and provide a hole injection barrier because of PC_61_BM's ([6,6]‐phenyl‐C61‐butyric acid methyl ester) low HOMO level, thereby decreasing reverse dark currents. Consequently, we fabricated ternary OPDs using PTB7‐Th, ICS, and PC_61_ BM with an optimized weight ratio of 1:1.5:1.5 (Table S1, Supporting Information). The absorption spectrum of the ternary device reveals the presence of PC_61_ BM (absorption peak at 355 nm) and minimal impact on the absorption in the NIR region (**Figure** **4**a). The reverse dark current density of ICS devices with PC_61_ BM is over one order of magnitude lower than the OPDs without PC_61_ BM, as shown in Figure 4b. However, the corresponding *R* values of ICS devices with PC_61_ BM do not show a significant reduction in the NIR region relative to devices without PC_61_ BM (Figure 4c). The details of device performance are outlined in **Table** **1**.

**Table 1:** The *J*_d_, *S*_n_, *R*, and corresponding *D* ^*^ of OPDs.

	*J*_d_ @ −0.2 V [A cm^−2^]	*S_n_* @ −0.2 V [A Hz^−1/2^]	*R* @ −0.2 V [A W^−1^]	D^*^ @ −0.2 V [Jones]
			950 nm	
Y6	1.52 × 10^−8^	3.09 × 10^13^	0.01	1.29 × 10^10^
ICS	2.36 × 10^−8^	2.64 × 10^13^	0.27	3.52 × 10^11^
ICS: PC_61_BM	1.23 × 10^−9^	1.32 × 10^14^	0.27	1.09 × 10^13^

According to Figure 4d, the PC_61_ BM OPD displayed a linear response across six orders of magnitude when exposed to a 950 nm LED, leading to a wide linear dynamic range (LDR) of 120 dB. In contrast, the device without PC_61_ BM displayed a smaller LDR of 101 dB, mainly attributed to its higher noise current.

The dark current only contributes to the shot noise, and neglecting other sources of noise could lead to an overestimation of the actual detectivity.^[^ ^27^ ^]^ Consequently, the devices’ noise current (Sn) was also measured to assess the device's detectivity accurately (Figure 4e; Figure S12, Supporting Information). As the modulated frequency increases, the measured *S*_n_ decreases. At −0.2 V, the measured *S_n_* at 169 Hz for devices with and without PC_61_ BM is 1.32 × 10^−14^ and 2.64 × 10^−13^ A Hz^−1/2^, respectively. As the modulated frequency increases further, the noise current of ternary OPDs reaches a plateau, which differs from the behavior observed in binary OPDs (Figure 4e). This phenomenon can be attributed to the reduced trap density in ternary OPDs containing PC_61_ BM, resulting in a rapid transition from 1/*f* noise to white noise. In contrast, the higher trap density in binary OPDs maintains the presence of 1/*f* noise. The calculated *D* ^*^ values (Equation S3, Supporting Information) at a −0.2 V for OPDs can be found in Figure 4f. The device with PC_61_ BM demonstrates *D* ^*^ values exceeding 1.0 × 10^12^ Jones from the wavelength of 350 to 980 nm, with a peak *D* ^*^ of 1.23 × 10^13^ Jones at 880 nm. Notably, the devices with PC_61_ BM maintain a *D* ^*^ of over 1.0 × 10^13^ Jones from 880 to 950 nm, ranking among the top NIR detectives in OPDs reported to date (Table 1; Table S2, Supporting Information).^[^ ^10^, ^28^, ^29^, ^30^, ^31^, ^32^ ^]^

**Figure 5:**
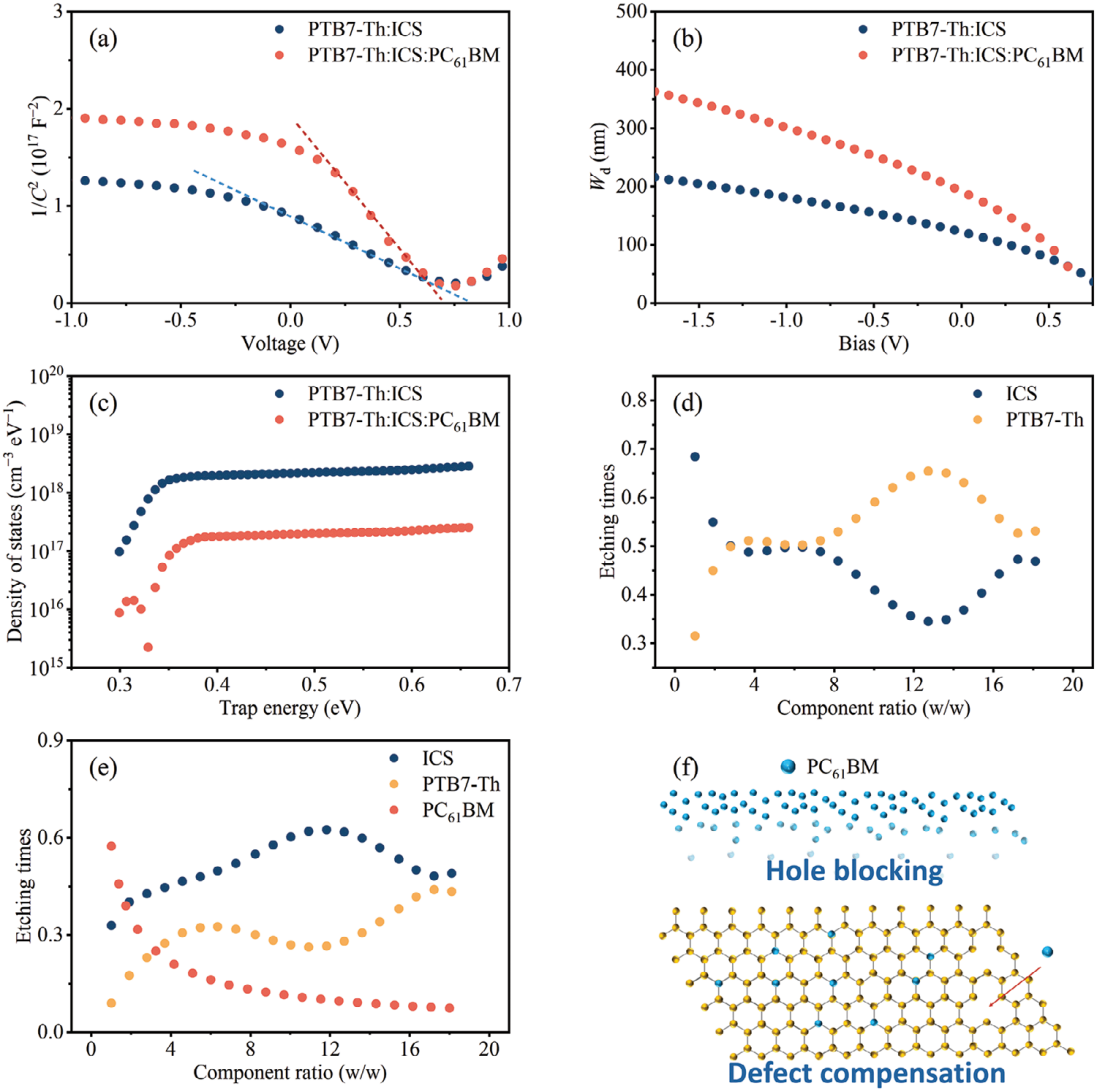
a) Mott–Schottky plots (dashed lines represent the linear fitting and b) *W_d_* −*V* curves of the optimized OPD devices based on ICS and ICS: PC_61_BM. c) Trap DOS. d) The Composition ratio of PTB7‐Th: ICS and e) PTB7‐Th: ICS: PC_61_ BM along the vertical direction of the active layer. f) Schematic Diagram of Blocking and Compensation Mechanisms of PC_61_BM.

To understand the mechanism behind the reduction in *J*_d_, capacitance‐voltage (*C*–*V*) measurements combined with Mott–Schottky analysis were conducted to examine the effects of PC_61_ BM addition on trap density and depletion width (**Figure** **5**; Figure S13, Supporting Information). For comparison, the thickness of the active layer for both the binary and ternary OPDs is tuned to be ≈180 nm. Mott–Schottky analysis reveals a depletion width of 125 nm for the binary OPD and 197 nm for the ternary OPD, respectively. These results indicate that both devices could achieve nearly full depletion with efficient charge collection (Equation S4, Supporting Information). Therefore, the analysis assumes that free carriers are entirely depleted in the space charge region at the p–n junction, with the charge in this area derived exclusively from dopant atoms or molecules.^[^ ^33^ ^]^ The Mott–Schottky plot, derived from *C* ^−2^–*V*, shows a linear relationship, where the slope indicates the trap density and the voltage axis intersection corresponds to the built‐in voltage (*V*_bi_). Incorporating PC_61_ BM into the device reduced the trap density (*N*_A_) from 1.84 × 10^16^ to 6.13 × 10^15^ cm^−3^ (Equation S5, Supporting Information).^[^ ^34^ ^]^ The ICS: PC_61_ BM device also exhibits a lower doping density and fewer non‐depleted regions than the ICS device. Additionally, to study the distribution of trap density of states (DOS) with PC_61_ BM integration, capacitance‐frequency (*C*–*ω*) measurements (Figure S13, Supporting Information) were carried out. A small AC bias is applied in this method to cyclically capture and release carriers from energy states close to the Fermi level.^[^ ^35^, ^36^ ^]^ As shown in Figure 5c, the trap DOS for the PC_61_BM‐integrated device is significantly lower than that of the device without PC_61_ BM, in the energy range of 0.35–0.60 eV, indicating reduced disorder in the film. This result is consistent with the lower *J*_d_ and higher *D* ^*^ observed in the former case compared to the latter.

The vertical composition distribution was analyzed through XPS depth profiling to investigate the composition from the air/active layer interface to the buried active layer/PEDOT: PSS/ITO interface. As the etching time increases, there is a noticeable decrease in the proportion of PC_61_ BM in the blend film (Figure 5e). This suggests that PC_61​_BM preferentially accumulates at the active layer/air interface of the BHJ film, rather than being uniformly distributed throughout the bulk of the film. Consequently, it can be inferred that PC_61_ BM concentrated within the active layer/ZnO interface of the BHJ film could effectively inhibit the leakage current resulting from hole migration, thereby diminishing *J*_d_ of the device. Besides, the incorporation of PC_61_ BM in the ternary system (Figure 5d) modulates vertical phase separation, resulting in a more uniform donor‐acceptor distribution when compared to the binary system (Figure 5e; Figure S14, Supporting Information). This uniformity reduces bimolecular charge recombination, leading to lower dark current and improved photocurrent and fill factor.^[^ ^37^ ^]^ Based on the above analysis and as illustrated in Figure 5f, the incorporation of PC_61_ BM serves dual roles: hole injection barrier and doping compensation, synergistically reducing dark current in OPDs.

The morphologies of the active layers in the two OPD devices were examined using atomic force microscopy (AFM). The height images of the PTB7‐Th: ICS and PTB7‐Th: ICS: PC_61_ BM blend films are presented in Figure S15 (Supporting Information), respectively. The incorporation of PC_61_ BM into the ICS blends results in slightly more uniform film morphology. The charge transporting properties of the PTB7‐Th: ICS and PTB7‐Th: ICS: PC_61_ BM blend films were investigated through the analysis of *J*−*V* plots in the dark for both electron‐only and hole‐only devices (Figure S16 and Table S3, Supporting Information). The electron mobility (µ_e_)/hole mobility (µ_h_) was estimated to be (8.1 ± 0.2) × 10^−5^ cm^2^ V^−1^ s^−1^/(6.6 ± 0.2) × 10^−5^ cm^2^ V^−1^ s^−1^ for the PTB7‐Th: ICS blend film and (2.2 ± 0.1) × 10^−4^ cm^2^ V^−1^ s^−1^ / (6.5 ± 0.1) × 10^−5^ cm^2^ V^−1^ s^−1^ for the PTB7‐Th: ICS: PC_61_ BM blend film. The PTB7‐Th: ICS: PC_61_ BM blend film demonstrates slightly higher electron mobility than the PTB7‐Th: ICS blend film.

**Figure 6:**
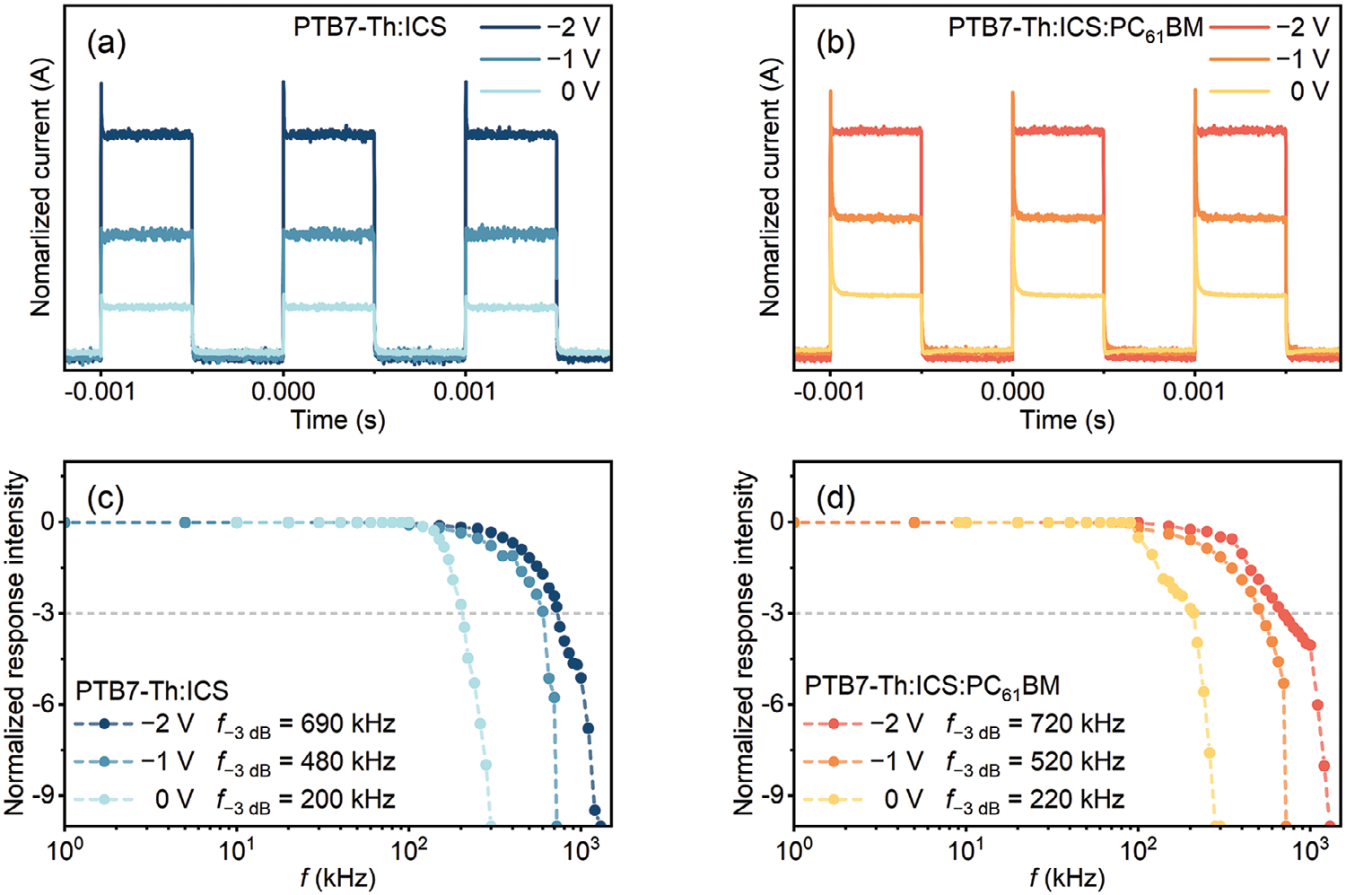
The normalized photocurrent of a) PTB7‐Th: ICS and b) PTB7‐Th: ICS: PC_61_ BM devices was measured under −0.2 V bias at a frequency of 2 kHz. Additionally, the cut‐off frequencies (*f*_−3_ _dB_) of the optimized OPD devices based on c) PTB7‐Th: ICS and d) PTB7‐Th: ICS: PC_61_ BM were determined under 0 V.

Response speed is an important parameter for OPDs. The transient photocurrent response was measured, and the response time was assessed by examining the photocurrent's rise and fall times as the light source was rapidly toggled on and off.^[^ ^38^ ^]^ As depicted in **Figure** **6**a,b, the photodetectors utilizing PTB7‐Th: ICS and PTB7‐Th: ICS: PC_61_ BM at −1 V demonstrate rapid response characteristics (*t*_rise_ is the time it takes for the response signal to rise from 10% to 90%, while *t*_fall_ is the time for it to drop from 90% to 10%), with *t*_rise_/*t*_fall_ of 0.73 µs/3.12 µs and 0.64 µs/1.13 µs, respectively. The cutoff frequency, a critical metric for assessing a photodetector's operational bandwidth, is defined as the frequency at which the device's output attenuates to −3 dB or 70.8% of its initial amplitude.^[^ ^39^ ^]^ Through comprehensive temporal photoresponse evaluations at varying light modulation frequencies under a bias of −2 V, the cutoff frequency (*f*_−3_ _dB_) for PTB7‐Th: ICS and PTB7‐Th: ICS: PC_61_ BM devices is 690 and 720 kHz (Figure 6c,d), respectively. Our photodetectors exhibit high response speed, outperforming most of the reported NIR OPD devices (>1 ms) (Table S2, Supporting Information).^[^ ^40^, ^41^, ^42^, ^43^, ^44^ ^]^ The response speed of a photodetector is mainly influenced by charge mobility and the *RC* time constant.^[^ ^45^ ^]^ Our device's mobility (2.2 × 10^−4^ cm^2^ V^−1^·s^−1^) is comparable to that of typical OPDs (10^−5^ to 10^−3^ cm^2^ V^−1^·s^−1^).^[^ ^46^, ^47^ ^]^ More importantly, the introduction of PC_61_ BM can reduce capacitance (Figure S13, Supporting Information), resulting in lower parasitic capacitance, thereby decreasing the *RC* time constant. Consequently, the charge extraction efficiency of OPDs can be enhanced with PC_61_ BM, enabling faster response times and a higher cutoff frequency.

**Figure 7:**
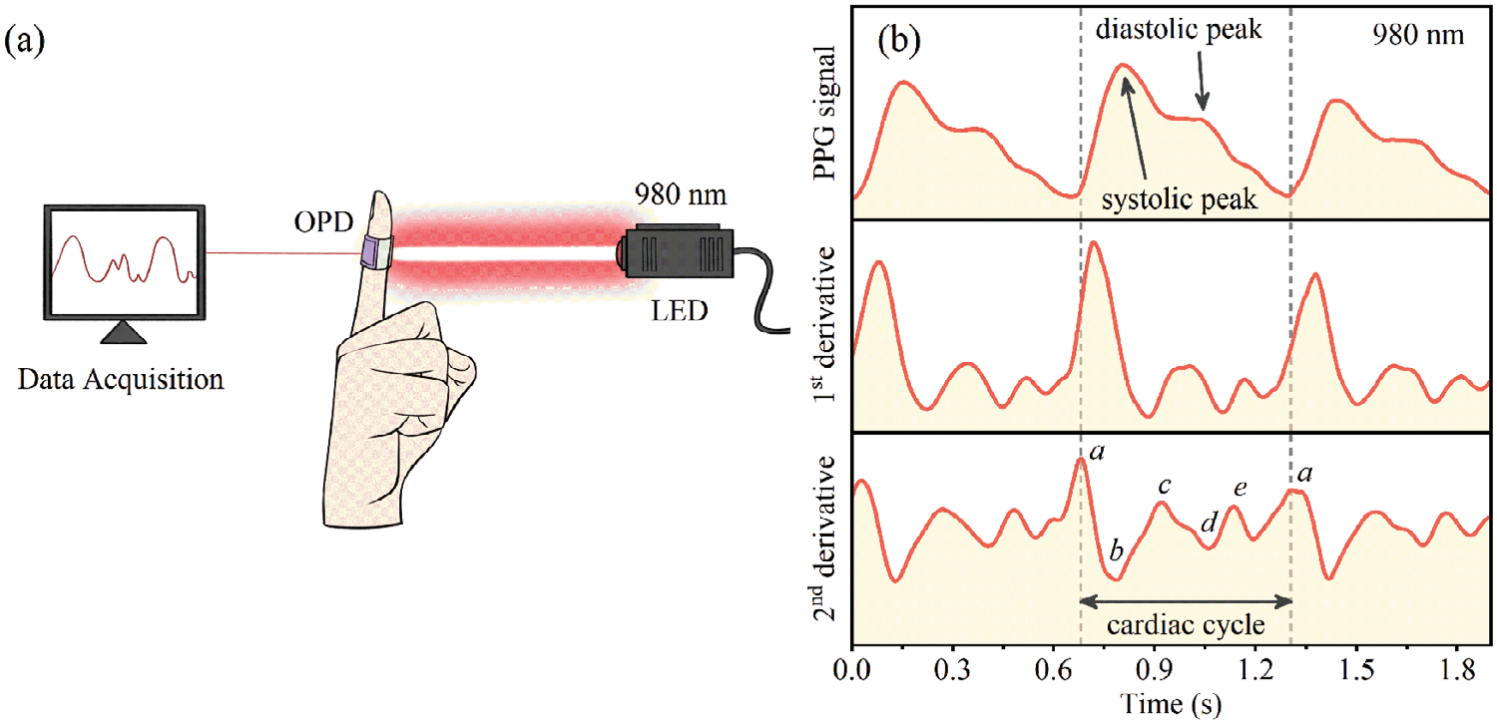
a) Schematic of the photoplethysmography (PPG) device and b) the pulse signals obtained from the OPD under 980 nm LED illumination through the fingertip.

The PPG signal captured blood volume fluctuations throughout the cardiac cycle, providing a detailed understanding of cardiovascular function.^[^ ^48^, ^49^ ^]^ As illustrated in **Figure** **7**a, We constructed a model PPG system that worked under transmission mode with a flexible OPD of PTB7‐Th: ICS: PC_61_ BM as the active layer and a 980 nm light‐emitting diode (LED). As depicted in Figure 7b, the heart rate was quantified to be 88 beats per minute for one of the contributing authors. The second derivative APG signal showed distinct systolic and diastolic waves throughout the cardiac cycle, offering valuable insights into cardiovascular health.^[^ ^50^ ^]^

## 3 Conclusion

In summary, a novel fused‐ring acceptor molecule, ICS, with an alkylthiol thiophene bridge, has been developed for NIR OPDs. ICS demonstrates an 80 nm redshift in the absorption peak compared to Y6, and it achieves nearly an order of magnitude reduction in dark current for OPDs when compared to devices using ICO molecules. Moreover, the introduction of a secondary acceptor, PC_61_ BM, in the active layer can reduce the reverse dark current of OPDs by decreasing trap density and acting as a hole barrier. Therefore, the device demonstrates a remarkable *D* ^*^ exceeding 10^13^ Jones in the 760–1000 nm range. By addressing the significant decline in *D* ^*^ at wavelengths beyond 900 nm—attributed to non‐radiative recombination losses and dark current noise—our devices improved *D* ^*^ by 2 to 3 orders of magnitude compared to most NIR OPDs. The device also demonstrates rapid response capabilities, with *t*_rise_ and *t*_fall_ values of 0.64 and 1.13 µs at −1 V, respectively. We overcame the reduction in response speed at higher frequencies due to limitations in carrier transport efficiency and device architecture, achieving a response speed over 100 times faster than most NIR OPDs. These accomplishments exceed the detection limits of commercially available silicon OPDs and highlight the potential of ICS‐based OPDs as promising alternatives to inorganic NIR OPDs. The optimized OPDs demonstrate the capability for real‐time heart rate monitoring utilizing PPG techniques. These findings confirm that ICS is an effective acceptor material for NIR OPDs. The enhanced performance of these devices facilitates their integration into flexible and wearable electronic systems with advanced functionalities.

## 4 Experimental Section

### Statistical Analysis

Data processing and analysis were conducted using Origin software, including normalization, fitting, and response analysis to comprehensively characterize the device's performance:

### Normalization

The absorption curve in Figure 2a was normalized to a range of 0–1 to enable comparison of relative absorption characteristics across samples.

### Fitting Analysis

Linear Dynamic Range (Figure 4d): The response data were linearly fitted to determine the device's response range under varying light intensities. Material Distribution (Figure 5d,e): Fitting was performed on the vertical composition distribution within the active layer to illustrate the effect of PC_61_ BM on material uniformity.

### Mott–Schottky Analysis

Linear fitting of *C* ^−2^–*V* curves (Figure 5a and **Table** **2**) was used to calculate *V*_bi_ and *N*_A_ ​, providing insights into the influence of PC_61_ BM doping on charge blocking and compensation.

**Table 2:** The parameters were derived from Mott–Schottky analysis.

Device	*V_bi_* [V]	*N_A_* [cm^−3^]	*W_d_* [nm, 0 V]
ICS	0.83	1.84 × 10^16^	125
ICS: PC_61_BM	0.68	6.13 × 10^15^	197

### Noise Current Analysis

*S*_n_ was measured at various frequencies, as shown in Figure 4e, to accurately calculate the *D* ^*^. The frequency‐dependent noise current reveals the transition from 1/*f* noise to white noise with PC_61_ BM incorporation, reflecting changes in trap density.

### Transient Photocurrent Response Analysis

Transient photocurrent measurements were used to determine the *t*_rise_ and *t*_fall_, with the *f*_‐3_ _dB_ calculated to define the device's bandwidth (Figure 6c,d). This analysis assesses the enhancement in response speed due to PC_61_ BM addition.

## Conflict of Interest

The authors declare no conflict of interest.

## Supporting information



Supporting Information

## Data Availability

The data that support the findings of this study are available from the corresponding author upon reasonable request.
