# Supplementary material for: Fast Near‐Infrared Organic Photodetectors with Enhanced Detectivity by Molecular Engineering of Acceptor Materials
Source: Adv Sci (Weinh). 2024 Nov 27;12(3):2410332. doi: 10.1002/advs.202410332 (PMC11744665; doi:10.1002/advs.202410332)
Supplement: Supplementary file 1 — Supporting Information [file ADVS-12-2410332-s001.docx]

Supporting Information

Fast near-infrared organic photodiodes with enhanced detectivity by molecular engineering of acceptor materials

Wentao Zhong, Xinyuan Wang, Wei Wang, Zhulu Song, Yirong Tang, Bulin Chen, Tingbin Yang*, and Yongye Liang*

W. Zhong, X. Wang, Z. Song, Y. Tang, B. Chen, T. Yang, Y. Liang

Department of Materials Science and Engineering, Southern University of Science and Technology, Shenzhen 518055, China
E-mail: yangtb@sustech.edu.cn; liangyy@sustech.edu.cn

T. Yang

Core Research Facilities, Southern University of Science and Technology, Shenzhen 518055, China

W. Wang
Experiment and Practice Innovation Education Center, Beijing Normal University, Zhuhai 519087, China

**I. Syntheses and characterizations**

*Materials*: [7,7-bis(2-ethylhexyl)-10-trimethylstannyl-3,11-dithiatricyclo[6.3.0.02,6]undeca-1(8), 2(6),4,9-tetraen-4-yl]-trimethylstannane (CPDT-SnMe_3_) was purchased from Guangzhou Yunsun Biotech Co. LTD. 2-(5,6-difluoro-3-oxoinden-1-ylidene) propanedinitrile (IC-2F) was purchased from Derthon OPV Co. LTD, while other reagents were purchased from J&K Scientific Co., bidepharm Co., TCI chemical Co. Tetrahydrofuran (THF), toluene, and chlorform (CF) used for reactions were purified by a solvent purification system (Innovative Technology, Inc.) before using. Detailed synthetic method of ICS was listed below

**Scheme S1.** Synthesis of **ICS**

1. **Synthesis of 1.** 3-((2-ethylhexyl)thio)thiophene (2 g, 8.76 mmol) and 50 ml THF was added to a 100 ml two-necked flask. N-Bromosuccinimide (NBS) (1.7g, 9.63 mmol) was dissolved in 25 ml THF and added to the flask through a dropping funnel. The mixture was stirred at room temperature (RT) for 12 hours and then quenched by water. After being extracted with ethyl acetate (EA, 80 ml) for three times, The organic phase was dried with MgSO_4_ and evaporated *in vacuo*. The crude product was subjected to column chromatography on silica gel with PE as eluent to afford **1** as a colorless oil (2.5g, 93%). ^1^H NMR (500 MHz, Chloroform-d) δ 7.25 (d, J = 5.7 Hz, 1H), 6.93 (d, J = 5.7 Hz, 1H), 2.83 (s, 2H), 1.51 – 1.31 (m, 5H), 1.30 – 1.18 (m, 4H), 0.87 (dt, J = 11.9, 7.0 Hz, 6H). ^13^C NMR (126 MHz, CDCl3) δ 133.70, 130.15, 125.90, 113.45, 39.57, 39.32, 32.14, 28.77, 25.38, 22.98, 14.14, 10.77.
2. **Synthesis of 2.** **1** (2 g, 6.5 mmol) and 50 ml THF was added to a 100 ml two-necked flask under an argon gas atmosphere. After cooling to -78 °C, 3.9 ml lithium diisopropylamide (7.8 mmol) was added dropwise. The mixture was stirred at -78 °C for an hour and then 0.5 ml DMF was added. Then the mixture was slowly warmed to RT. After 12 hours, the reaction was quenched by water. After being extracted with ethyl acetate (EA, 80 ml) for three times, The organic phase was dried with MgSO_4_ and evaporated *in vacuo* without further purification.
3. **Synthesis of 3.** To a solution of the crude product (1.4 g, 4.2 mmol) and CPDT-SnMe_3_ (1 g, 1.4 mmol) in toluene (15 mL) under an argon gas atmosphere, Pd(PPh_3_)_4_ (80 mg) was added. The mixture was stirred at 110 °C for 24 hours. After cooling to room temperature, the solvents were evaporated in vacuo. The crude product was subjected to column chromatography on silica gel with PE/DCM 1:1 as eluent to afford **3** as a red solid. (778 mg, 61%) ^1^H NMR (500 MHz, Chloroform-*d*) δ 9.81 (s, 2H), 7.68 (s, 2H), 7.44 (t, *J* = 2.8 Hz, 2H), 2.93 – 2.89 (m, 4H), 2.03 – 1.87 (m, 6H), 1.49 – 1.40 (m, 8H), 1.03 – 0.82 (m, 37H), 0.71 (dt, *J* = 7.0, 3.4 Hz, 8H), 0.63 (td, *J* = 7.3, 2.4 Hz, 6H). ^13^C NMR (126 MHz, CDCl_3_) δ 182.03, 158.79, 146.49, 141.85, 140.83, 138.76, 135.67, 130.03, 129.07, 123.19, 54.21, 43.17, 41.20, 39.62, 35.43, 34.40, 32.36, 29.92, 29.85, 29.70, 29.67, 29.63, 29.47, 29.39, 29.26, 28.88, 28.69, 27.57, 27.36, 25.65, 23.11, 22.93, 22.84, 14.26, 10.91, 10.85.
4. **Synthesis of ICS.** IC-2F (100 mg, 0.43 mmol) and 25 ml CF was added to a 100 ml two-necked flask under an argon gas atmosphere. Then pyridine (0.3 ml) was added dropwise to initiate the reaction and the solution was immediately changed into red. The mixture was stirred at RT for an hour. After **3** (100 mg, 0.1mmol) was dissloved in 10 ml CF and added dropwise, the mixture was stirred at 65 °C for 24 hours, and then cooled to RT. After the solvents were evaporated in vacuo, the crude product was subjected to column chromatography on silica gel with PE/DCM 1:2 as eluent to afford **ICS** as a dark green solid. (213.6 mg, 40%) ^1^H NMR (400 MHz, Chloroform-*d*) δ 8.73 (s, 2H), 8.55 (dd, *J* = 9.9, 6.5 Hz, 2H), 7.80 – 7.66 (m, 6H), 3.00 (dt, *J* = 6.2, 1.7 Hz, 4H), 2.01 (p, *J* = 9.1 Hz, 4H), 1.64 (dt, *J* = 12.3, 6.2 Hz, 3H), 1.47 (dp, *J* = 20.9, 7.1, 6.5 Hz, 8H), 1.30 (s, 12H), 1.09 – 0.86 (m, 31H), 0.72 (t, *J* = 6.2 Hz, 9H), 0.66 (t, *J* = 6.8 Hz, 6H). ^13^C NMR (101 MHz, CDCl_3_) δ 186.32, 160.75, 152.33, 149.86, 143.40, 137.34, 136.51, 133.72, 131.94, 124.28, 124.19, 121.70, 115.27, 115.06, 114.30, 112.89, 112.68, 54.33, 43.46, 41.22, 39.63, 35.53, 34.21, 32.48, 29.47, 28.93, 28.63, 27.52, 25.76, 23.11, 22.99, 14.22, 10.99, 10.80. HRMS(ESI) calcd for C_75_H_79_F_4_N_4_O_2_S_6_^+^, ([M+H]^+^) 1335.44634, Found 1335.44211

**
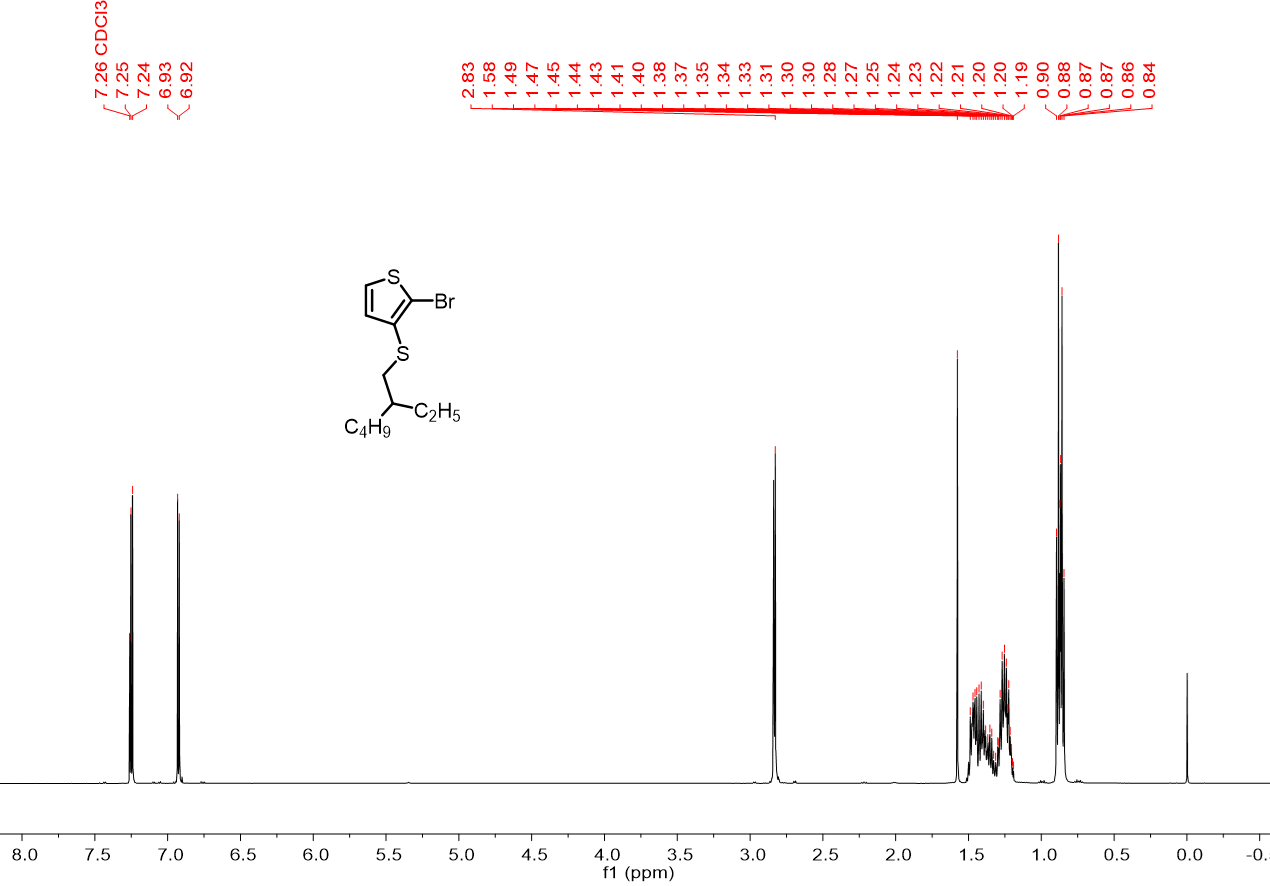
**

**Figure S1. ^1^H NMR spectrum of 1.**

**
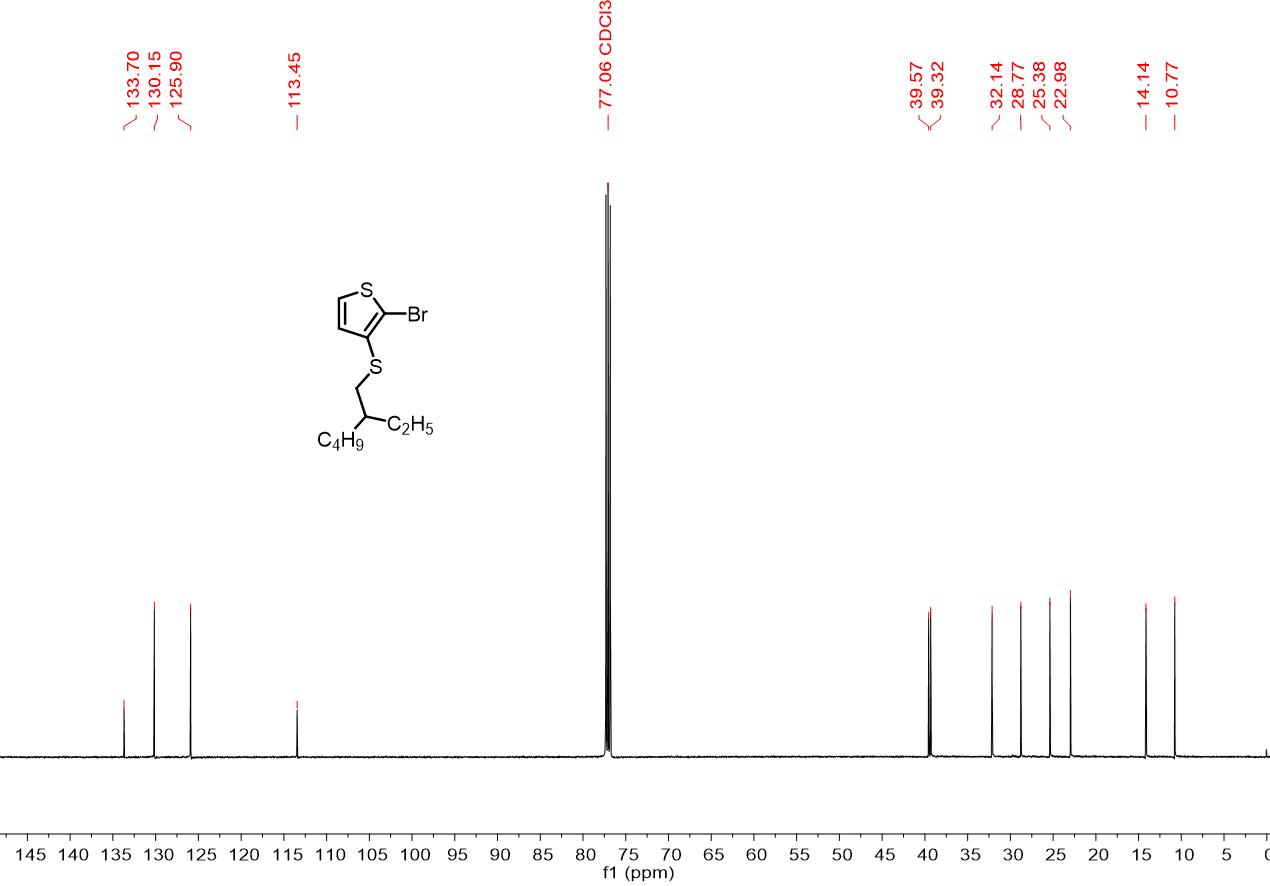
**

**Figure S2. ^13^C NMR spectrum of 1.**

**
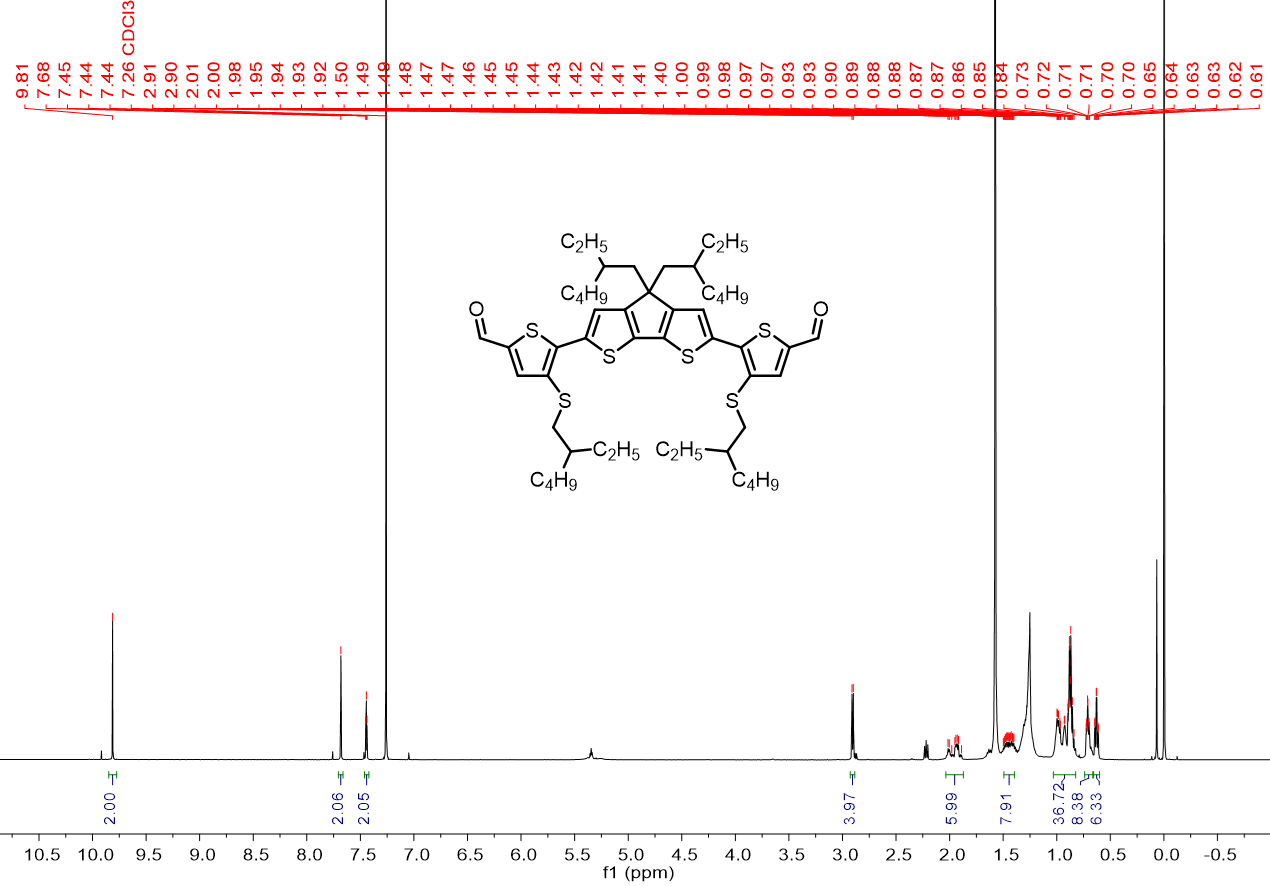
**

**Figure S3. ^1^H NMR spectrum of 3.**

**
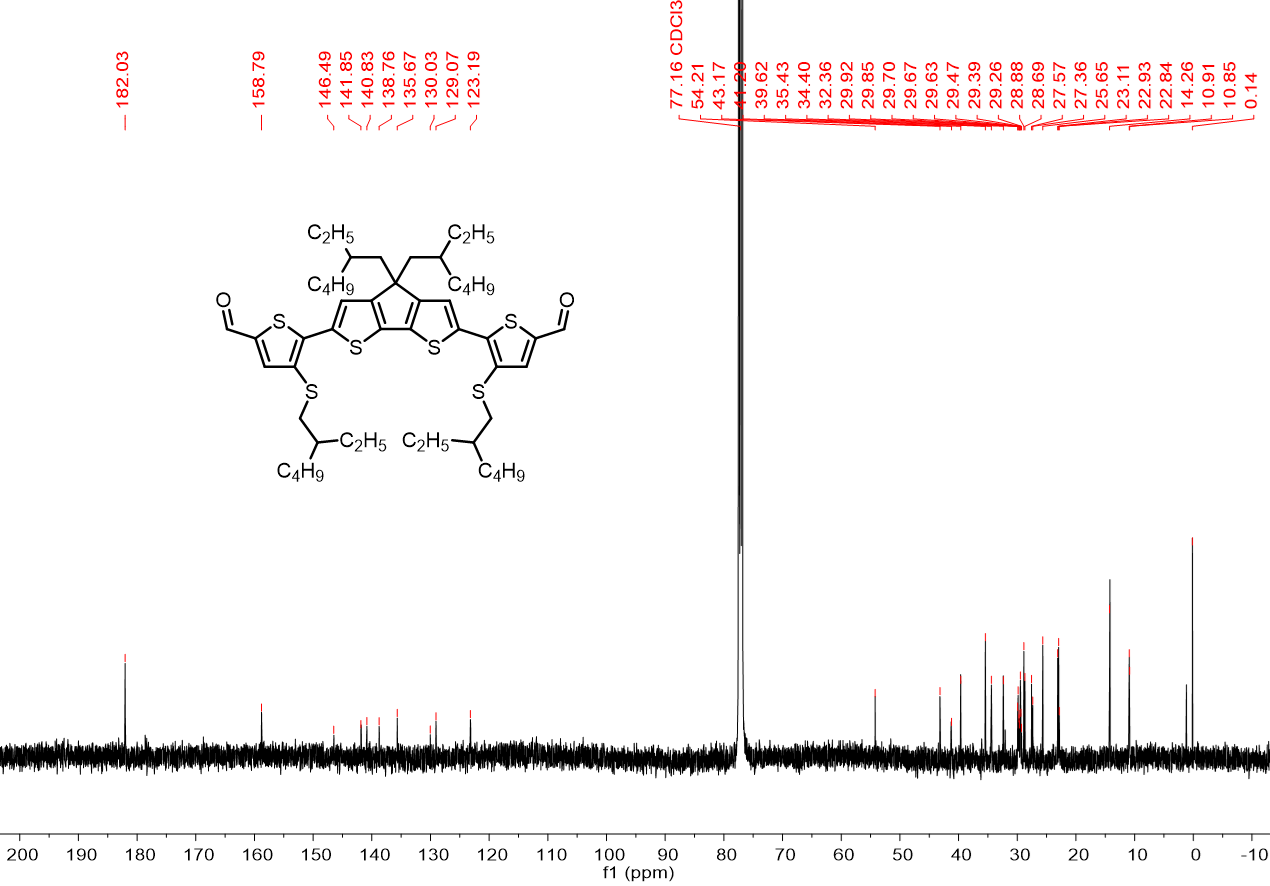
**

**Figure S4. ^13^C NMR spectrum of 3.**

**

**

**Figure S5. ^1^H NMR spectrum of ICS.**

**

**

**Figure S6. ^13^C NMR spectrum of ICS.**

**
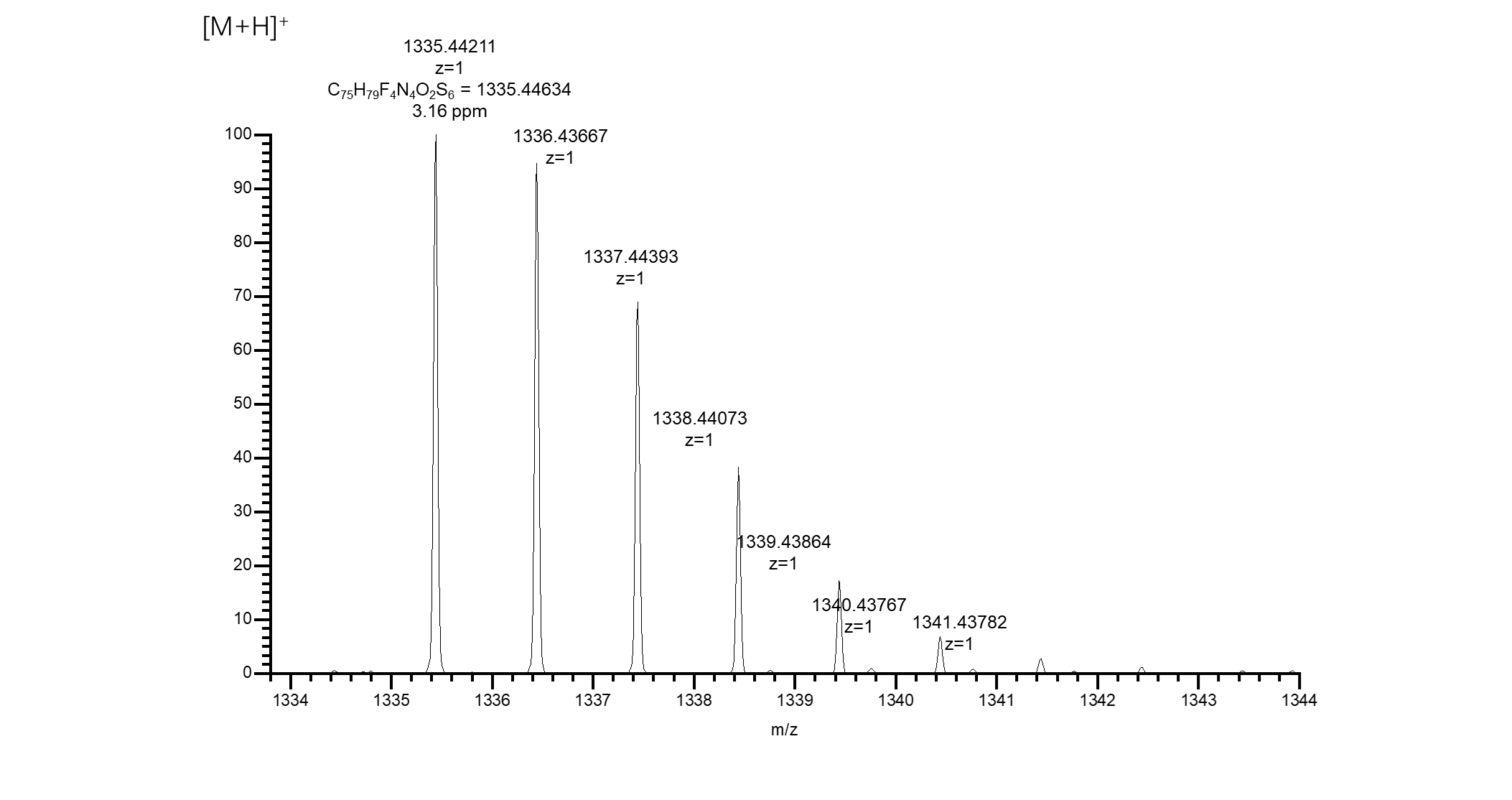
**

**Figure S7. HRMS of ICS.**

**II.** **Device Details**

*Materials*: PTB7-Th was purchased from 1-Material Inc. Y6 was purchased from eFlexPV Limited (China). The PEDOT: PSS solution was purchased from Heraeus. The ZnO NCs (12 nm) were purchased from Avantama AG. [6,6]-Phenyl-C_61_-butyric acid methyl ester (PC_61_BM, ≥99.5%) was obtained from American Dye Source, Inc. (Quebec, Canada). Chloroform (anhydrous, Aladdin) was purified by a solvent purification system (Innovative Technology, Inc.) before device fabrication. 1,2-Dichlorobenzene (DCB, anhydrous) was purchased from Sigma-Aldrich, Inc.

*Device fabrication*: The ITO substrates were subsequently sonicated with deionized water, acetone, and isopropanol for 30 minutes, and then dried in the oven. Next, the substrate was treated with UVO for 15 minutes in a UV-ozone chamber. Then, PEDOT: PSS was spin-coated on top to form a thin layer (⁓25 nm) and annealed for 15 minutes at 150°C. For binary devices, PTB7-Th, Y6, or ICS were blended with a weight ratio of 1:1.5 and dissolved in chloroform with 2% 1-chloronaphthalene. Then, an active layer with a thickness of 180 nm was spun onto the substrates. After annealing the active layer at 100°C for 10 minutes, a thickness of 10 nm ZnO NCs was spun onto it. Finally, a 120 nm Al layer was deposited by thermal evaporation under a vacuum of 2×10^−6^ torr. For ternary devices, PTB7-Th, ICS, and PC_61_BM were blended with a weight ratio of 1:1.5:1.5 and dissolved in chloroform with 2% 1-chloronaphthalene and 10% 3,3-dichlorobenzidine. The following procedures were the same as in binary devices.

*Device characterization*: The absorption spectrum was measured with a UV-Vis-IR spectrophotometer (Shimadzu, UV3600). Cyclic voltametry (CV) measurements were carried out using an electronchemical workstation (CHI600E) to determine the HOMO and LUMO levels of all materials. The *J*−*V* measurement of the devices under AM 1.5 G solar simulator illumination (100 mW cm^−2^) was performed using a computer-controlled Keithley 2400 Source Measure Unit in ambient. The *J*−*V* characteristics of the devices in dark were measured on a computer-controlled Keithley 2635 Source Measure Unit in ambient. The EQE was measured at room temperature under ambient atmosphere using an Enlitech QE−R system. A bromine tungsten lamp served as the light source. The atomic force microscopy (AFM) images of the active layer were measured with a multimode microscopy (Bruker, Inc.) and conducted at tapping mode condition. The noise current of the devices was measured using an FS−Pro Semiconductor Parameter Tester (Primarius), with a low-noise current preamplifier (SR830) and a lock-in amplifier in a dark chamber at ambient condition. The noise spectral density (*S*n(*f*)) was recorded from 1 Hz to 1 kHz, with each frequency point stabilized for 10 seconds prior to acquisition. Calibration was conducted using a silicon reference device to ensure accuracy, and background noise was subtracted. The total noise current, calculated by integrating *S*n(*f*) over the frequency range, was 1.33 × 10^−11^ A for the device without PC_61_BM and 3.25 × 10^−12^ A for the device with PC_61_BM. Measurements were repeated three times for consistency. X-ray photoelectron spectroscopy (XPS) depth profiling of the active layers was conducted on devices with the structure active layer/PEDOT: PSS/ITO, using an ESCALAB 250 (Thermo-VG Scientific). The samples underwent sputtering with an Ar+ gun at 3 kV and 2 μA, with etching initiated from the air/active layer interface. The XPS depth profile scanning covered an area of 2 mm × 2 mm, while composition data were collected from a region measuring 0.5 mm × 0.2 mm. Capacitance–voltage (*C*–*V* ) curve was carried out for Mott–Schottky analysis by an impedance spectroscope (Paios LiquidNitrogen, FLUXiM) with a frequency of 10 kHz. The fabricated OPDs were evaluated using a SOFN7SCF06A/B probe station in conjunction with a Keithley 4200 measurement system. Illumination was provided by a Zolix TLS2-T250-DZL halogen light source, and the light intensity was measured using a Thorlabs PM 100D power meter equipped with appropriate OPDs. For frequency response characterization, the incident light was modulated using an SR540 light chopper.


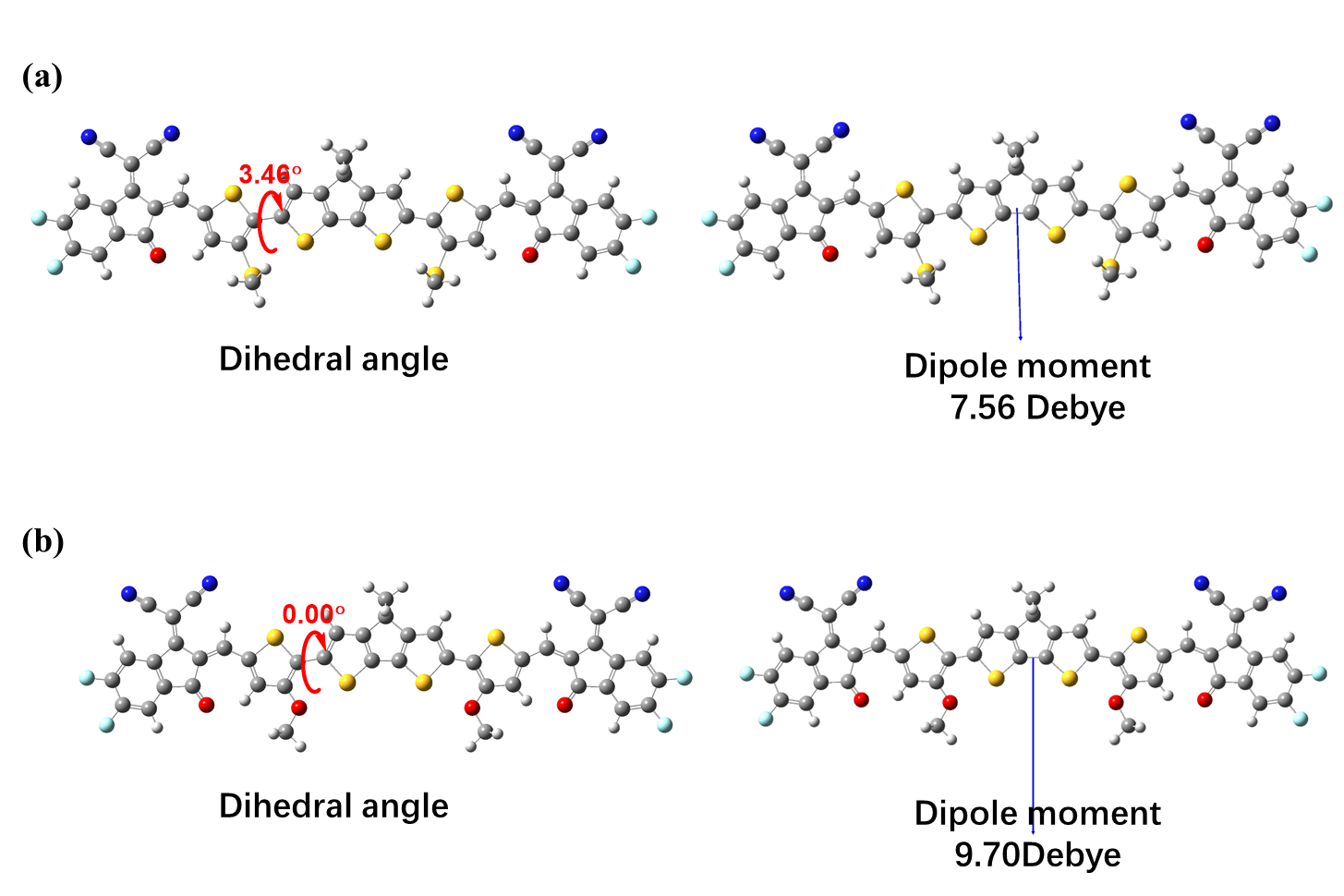


**Figure S8.** The geometry-optimized structures for the model molecule of (a) ICS and (b) ICO (alkyl chains were simplified as methyl groups).


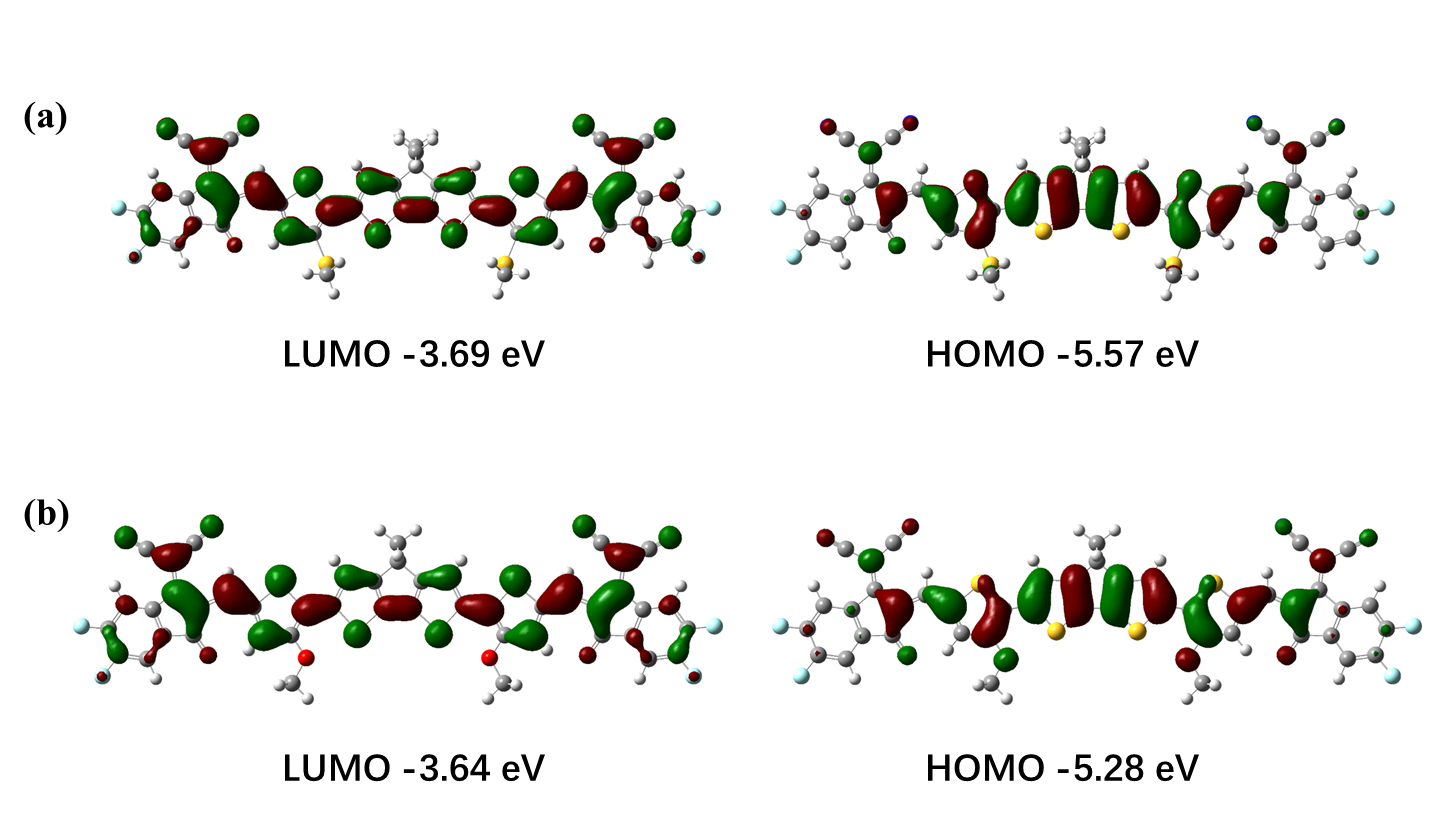


**Figure S9.** The main orbital configurations and electronic transitions of (a) ICS and (b) ICO for the main absorption band based on the TD-DFT calculations.

**

**

**Figure S10.** (a) Absorption spectra of ICS and ICO in thin films. (b) *J*−*V* curves were measured in darkness for the PTB7-Th devices.

**

**

**Figure S11.** Cyclic voltammogram of ICS for the positive scan (a) and negative scan (b). The redox potential of ferrocene/ferrocenium (Fc/Fc+) under the same conditions is located at 0.5 V, (c) which is assumed to have an absolute energy level of -4.8 eV to vacuum.





**Figure S12.** The noise spectral currents of (a) PTB7-th: ICS and (b) PTB7-th: ICS: PC_61_BM based OPD devices.

**

**

**Figure S13.** (a) *C*–*V* characteristics. (b) *C*–*ω* curves.





**Figure S14.** The correlation between the distribution of PC_61_BM concentration in the active layer and etching durations.

| **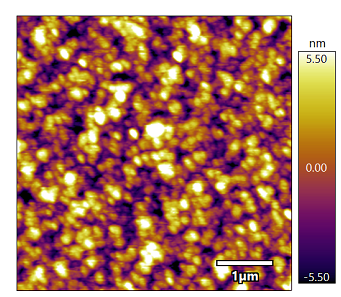**  **(a)** | **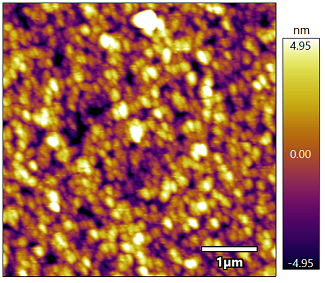**  **(b)** | **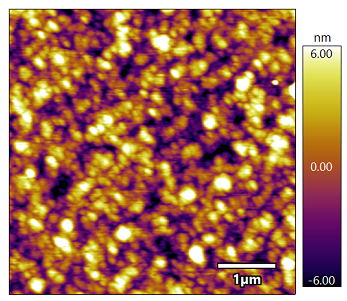**  **(c)** |
| --- | --- | --- |
| **Rq= 2.74 nm** | **Rq= 2.99 nm** | **Rq= 2.47 nm** |
|  |  |  |

**Figure S15.** The AFM height images of (a) PTB7-Th: ICS ,(b) PTB7-Th: ICS: PC_61_BM blend films, and (c) PTB7-Th: ICO.





**Figure S16**. Hole and electron mobilities of acceptor only films and blend films.

**Table S1.** Performance of ICS-based devices at various PTB7-Th: ICS: PC_61_BM ratios.

| Ratio  [w/w] | *J*_d_ @ -0.2 V  [A cm^-2^] | *R*@ 880 nm  [A W^-1^] | Response time @ -0.2 V  [μs] |
| --- | --- | --- | --- |
| 1:1.2 | (2.44 $\pm$ 0.2) ×10^-5^ | 0.23 $\pm$ 0.04 | 1.1 $\pm$ 0.2 |
| 1:1.5 | (1.20 $\pm$ 0.2) ×10^-6^ | 0.36 $\pm$ 0.02 | 1.0 $\pm$ 0.4 |
| 1:2 | (1.52 $\pm$ 0.3) ×10^-6^ | 0.30 $\pm$ 0.02 | 2.2 $\pm$ 0.2 |
| 1:1.5:0.2 | (8.68 $\pm$ 0.2) ×10^-7^ | 0.35 $\pm$ 0.02 | 1.2 $\pm$ 0.4 |
| 1:1.5:0.5 | (6.27 $\pm$ 0.2) ×10^-7^ | 0.35 $\pm$ 0.02 | 1.2 $\pm$ 0.4 |
| 1:1.5:1 | (3.24 $\pm$ 0.2) ×10^-7^ | 0.35 $\pm$ 0.03 | 1.2 $\pm$ 0.3 |
| 1:1.5:1.5 | (6.93 $\pm$ 0.2) ×10^-8^ | 0.34 $\pm$ 0.03 | 1.0 $\pm$ 0.4 |
| 1:1.5:2 | (6.84 $\pm$ 0.2) ×10^-8^ | 0.33 $\pm$ 0.04 | 1.1 $\pm$ 0.4 |
| 1:1.5:2.5 | (6.53 $\pm$ 0.3) ×10^-8^ | 0.32 $\pm$ 0.04 | 1.2 $\pm$ 0.4 |

**Table S2.** Comparison of organic infrared detectors, with response to NIR.

|  | Range  [nm] | *J*_d_  [nA cm^2^] | *R*  [A W^-1^] | *D*^*^  [Jones] | τ_rise_/τ_fall_ | *f*-3dB  [kHz] | Reference |
| --- | --- | --- | --- | --- | --- | --- | --- |
| PCE10:CO1-4Cl | 300-960 | 7.0 @ -2 V | 0.5 | 3.31 × 10^13^ |  | 240 | ^[1]^ |
| Polymer:PCBM | 330–950 | 1.1 @ -0.1 V | 0.06 | 1.4×10^12^ |  |  | ^[2]^ |
| PCE10:BDP4Cl | 300–1300 | 0.36 @ 0 V | 0.17 | 3.81×10^12^ | 8.14/8.18 μs |  | ^[3]^ |
| PIPCP:PCBM | 410-800 | >10^5^ |  |  | 0.2 μs | 1 | ^[4]^ |
| PCE10:COTIC | 780–1070 | 8.6 @ -2 V | 0.45 | 10^13^ | 8/8 μs |  | ^[5]^ |
| TbzIC: PBDT-TT | 500-1300 | 0.24 @ 0 V | 0.37 | 10^13^ | 20/10 μs |  | ^[6]^ |
| PBIBDF-BT | 600-1000 |  | 0.02 | ~4 × 10^11^ | 20/30 ms |  | ^[7]^ |
| PMDPP3T:PCBM | <850 | 100 @ -2 V | 0.57 | 3.2× 10^11^ | 150 μs |  | ^[8]^ |
| P3HT:PCBM | 700-1300 |  | 0.4 | 3.2 × 10¹¹ |  |  | ^[9]^ |
| PIF:ITIC | 600-800 | 10 @ -2 V | 0.23 | 6.9×10^10^ | 2.5μs | 1500 | ^[10]^ |
| PCE10:COTIC | 400-1100 | 1.2 @ -0.1V | 0.41 | 2.1×10^13^ |  | 45 | ^[11]^ |
| PBTI(EDOT):PC_71_BM | 400-1085 | 6.7 @ -0.2V |  | 1.8×10^12^ |  |  | ^[12]^ |
| PTB7-Th:ICS:PC_61_BM | 300-1100 | 1.23 @ -0.2V | 0.27 | 1.09×10^13^ | 0.64 /1.13 μs | 720 | This work |

**Table S3.** Hole and electron mobilities of the blend films.

|  | μ_h_  [cm^−2^ V^−1^ s^−1^] | μ_e_  [cm^−2^ V^−1^ s^−1^] | μ_e_/μ_h_ |
| --- | --- | --- | --- |
| ICS: PC_61_BM | 6.5×10^−5^ | 2.2×10^−4^ | 3.66 |
| ICS | 6.6×10^−5^ | 8.1×10^−5^ | 1.34 |

**Parameters and Equations**

*R* represents the response of the photodetector to a specific wavelength, which is defined as the ratio of the photocurrent output to the optical input power:

$R=\frac{J_{ph}}{P_{in}}=\frac{EQE\times\lambda\times q}{h\times c}$ (1)

where *J*_ph_ is the photocurrent density, *P*_in_ is the incident light intensity, *λ* is the wavelength of incident light, *q* is the elementary charge, *h* is the Planck constant, and c is the speed of light.

Specific detectivity (*D**) is an important figure-of-merit for a photodetector, used to assess its sensitivity in detecting weak light signals. Assuming that shot noise is the primary source of electronic noise in devices, *D** can be approximately calculated using the following equation:

${D_{sh}}^{*}=\frac{R\sqrt{A}}{\sqrt{2qi_{d}}}=\frac{R}{\sqrt{2qJ_{d}}}$ (2)

where *q* is the elementary charge and ​ *J*_d_ is the dark current. The dark current is a critical parameter for a photodetector, as it plays an important role in determining the maximum limit of light detection capability.

For a more precise calculation, *D** is determined by the device noise current and responsivity (*R*), which can be expressed as:

$D^{*}=\frac{R\sqrt{AB}}{i_{n}}$ (3)

where *A* is the effective area of the device, *B* is the bandwidth, and *i*_n_ is the noise current.

The depletion width (*W*), also referred to as the space charge width, can be expressed as:

$W=\sqrt{\frac{2\varepsilon_{r}\varepsilon_{0}\left( V_{bi}-V \right)}{qN_{A}}}$ (4)

where *W* is a function of the trap density ($N_{A}$​), built-in voltage (*V*_bi_), and the applied voltage (*V*). *W* plays a crucial role in determining the charge transport properties of the device. The slope of the *C*^−2^-*V* plot provides insight into the trap density, while the intercept with the voltage axis yields the *V*_bi_. $N_{A}$ is quantitatively defined by the following expression:

$N_{A}=\frac{-2}{q\varepsilon_{r}\varepsilon_{0}A^{2}}\left( \frac{dV}{dC^{2}} \right)$ (5)

where $\varepsilon_{r}$ is the relative dielectric constant of the BHJ film (assuming $\varepsilon_{r}$ = 3), $\varepsilon_{0}$ is the vacuum permittivity, and A is the area of the device, the value of $N_{A}$ is negatively correlated with the dark current. the $N_{A}$ values for the ICS-based device and the ICS: PC_61_BM-based device are 1.84×10^16^ cm^-3^ and 6.13×10^15^ cm^-3^, respectively.

**Supplementary references**

[1] J. Huang, J. Lee, J. Vollbrecht, V.V. Brus, A.L. Dixon, D.X. Cao, Z. Zhu, Z. Du, H. Wang, K. Cho, *Adv. Mater.* **2020**, *32*, 1906027.

[2] J. Qi, X. Zhou, D. Yang, W. Qiao, D. Ma, Z.Y. Wang, *Adv. Funct. Mater.* **2014**, *24*, 7605.

[3] M. Yang, B. Yin, G. Hu, Y. Cao, S. Lu, Y. Chen, Y. He, X. Yang, B. Huang, J. Li, *Chem* **2024**, *10*, 1425.

[4] S. Park, K. Fukuda, M. Wang, C. Lee, T. Yokota, H. Jin, H. Jinno, H. Kimura, P. Zalar, N. Matsuhisa, *Adv. Mater.* **2018**, *30*, 1802359.

[5] Y. Song, Z. Zhong, P. He, G. Yu, Q. Xue, L. Lan, F. Huang, *Adv. Mater.* **2022**, *34*, 2201827.

[6] B. Yin, X. Zhou, Y. Li, G. Hu, W. Wei, M. Yang, S. Jeong, W. Deng, B. Wu, Y. Cao, *Adv. Mater.* **2024**, *36*, 2310811.

[7] G. Wang, K. Huang, Z. Liu, Y. Du, X. Wang, H. Lu, G. Zhang, L. Qiu, *ACS Appl. Mater. Interfaces* **2018**, *10*, 36177.

[8] T. Yokota, T. Nakamura, H. Kato, M. Mochizuki, M. Tada, M. Uchida, S. Lee, M. Koizumi, W. Yukita, A. Takimoto, *Nat. Electron.* **2020**, *3*, 113.

[9] P.C. Chow, T. Someya, *Adv. Mater.* **2020**, *32*, 1902045.

[10] N. Strobel, N. Droseros, W. Köntges, M. Seiberlich, M. Pietsch, S. Schlisske, F. Lindheimer, R.R. Schröder, U. Lemmer, M. Pfannmöller, *Adv. Mater.* **2020**, *32*, 1908258.

[11] C. Xu, P. Liu, C. Feng, Z. He, Y. Cao, *J. Mater. Chem. C* **2022**, *10*, 5787.

[12] L. Zhang, T. Yang, L. Shen, Y. Fang, L. Dang, N. Zhou, X. Guo, Z. Hong, Y. Yang, H. Wu, *Adv. Mater. (Deerfield Beach, Fla.)* **2015**, *27*, 6496.
